# Lower neurovascular coupling response despite higher cerebral blood flow at rest in apolipoprotein ɛ4 positive adults

**DOI:** 10.1371/journal.pone.0314744

**Published:** 2024-12-03

**Authors:** Andrew G. Pearson, Kathleen B. Miller, Adam T. Corkery, Nicole A. Loggie, Anna J. Howery, Leonardo A. Rivera-Rivera, Oliver Wieben, Kevin M. Johnson, Sterling C. Johnson, Jill N. Barnes

**Affiliations:** 1 Department of Kinesiology, Bruno Balke Biodynamics Laboratory, University of Wisconsin-Madison, Madison, WI, United States of America; 2 Department of Health and Exercise Science, University of St. Thomas, St. Paul, MN, United States of America; 3 Alzheimer’s Disease Research Center, University of Wisconsin-Madison School of Medicine and Public Health, Madison, WI, United States of America; 4 Department of Medical Physics, University of Wisconsin School of Medicine and Public Health, Madison, WI, United States of America; 5 Department of Radiology, University of Wisconsin School of Medicine and Public Health, Madison, WI, United States of America; Universitatsklinikum Regensburg, GERMANY

## Abstract

Cerebral blood flow at rest declines with age. However, age-related changes in functional measures of cerebrovascular health including cerebrovascular reactivity and neurovascular coupling are not well understood. Additionally, the effect of apolipoprotein E (*APOE*) ε4, a strong genetic risk factor for Alzheimer’s disease, on cerebral blood flow and cerebrovascular function remains unclear. *APOE*ε4 positive (*APOE*ε4+; n = 37, age = 63±4y) and *APOE*ε4 negative (*APOE*ε4-; n = 50, age = 63±4y) cognitively unimpaired adults participated in this study. Macrovascular cerebral blood flow and microvascular cerebral perfusion were measured using 4D flow MRI and pseudo-continuous arterial spin labeling MRI, respectively. Cerebrovascular reactivity and neurovascular coupling were assessed by measuring middle cerebral artery blood velocity in response to hypercapnia and the *n*-back test, respectively. Neurovascular coupling was lower in *APOE*ε4+ compared with *APOE*ε4- adults (*P*<0.05), despite higher cerebral blood flow and cerebrovascular reactivity to hypercapnia. Alterations in neurovascular coupling may occur early, prior to changes in cognition, in aging *APOE*ε4 carriers.

## Introduction

The apolipoprotein E (*APOE*) ε4 allele increases the risk of Alzheimer’s disease (AD) such that *APOE*ε4 carriers have a 2 to 15 times greater risk of developing AD [1,2]. However, although there is a strong influence of *APOE*ε4 on AD risk, *APOE*ε4 positivity does not guarantee development of AD [3]. As such, additional risk factors may provide enhanced insight for *APOE*ε4 carriers at genetic predisposition to develop mild cognitive impairment (MCI) and AD [4].

Alterations in cerebral blood flow (CBF) may occur prior to significant accumulation of AD neuropathology, changes in brain structure, and manifestation of AD symptoms [5,6]. Indeed, cross-sectional [7,8] and longitudinal [9,10] studies indicate a decline in CBF with advancing age, which is likely mediated by *APOE* genotype [11–13]. For example, CBF at rest is greater in *APOE*ε4 positive (*APOE*ε4+) compared with *APOE*ε4 negative (*APOE*ε4-) young adults yet lower in *APOE*ε4+ compared with *APOE*ε4- older adults [13]. These findings, as well as others [14–16], suggest a cerebrovascular compensation hypothesis which proposes a biphasic relationship between *APOE*ε4 and CBF across the lifespan, complicating the use of CBF at rest as an AD risk factor in aging adults. Functional measures of cerebrovascular health, in addition to CBF at rest, may provide insight into AD risk in *APOE*ε4 carriers across the lifespan.

Cerebrovascular function, or the cerebral blood flow or velocity response to chemical or cognitive stimuli, may be differentially affected by *APOE* genotype [17]. Cerebrovascular reactivity (CVR) to elevated carbon dioxide (hypercapnia) is a commonly used functional measure of cerebrovascular health. Previous research suggests a decline in CVR across the lifespan [8,18], with direct comparisons demonstrating lower CVR in older adults compared with young adults [19–21]. As a result, CVR has been hypothesized as a potential diagnostic tool to detect vascular dysfunction prior to declines in cognitive function in individuals at risk of MCI and AD [22]. Limited previous work suggests that CVR is lower in *APOE*ε4+ compared with *APOE*ε4- young [23] and older adults [24]; however, data in healthy, cognitive unimpaired adults in the 55–69 age range are lacking.

Another functional measure of cerebrovascular health is neurovascular coupling (NVC). NVC is the tight coupling between neuronal activity and CBF to maintain delivery of oxygenated blood and nutrients to the brain [25]. Impairments in NVC may be an early indicator of cerebrovascular dysfunction [26]. Consistent with the idea of cerebrovascular compensation in *APOE*ε4 carriers, *APOE*ε4+ young adults demonstrate augmented blood oxygen level dependent (BOLD) responses to memory tasks compared with *APOE*ε4- young adults [14,27]; however, similar studies in aging adults report mixed findings [28]. Importantly, many studies that assess the impact of *APOE*ε4 on functional measures of cerebrovascular health do not include tests that assess cerebrovascular responses to both chemical and cognitive stimuli. Furthermore, data in aging participants without multiple comorbidities such as poor vascular or cognitive health is lacking.

As vascular dysfunction may manifest prior to AD symptoms, it is possible that changes in cerebrovascular function occur earlier in *APOE*ε4+ carriers. Therefore, the purpose of this study was to comprehensively evaluate the influence of the *APOE*ε4 allele on both resting CBF and cerebrovascular function (measured by CVR and NVC) in cognitively unimpaired aging adults. We hypothesized that, compared with *APOE*ε4- adults, *APOE*ε4+ adults would demonstrate greater CBF at rest; however, they would demonstrate reduced responses to measures of cerebrovascular function including attenuated CVR to hypercapnia and impaired NVC in response to an acute cognitive challenge.

## Materials and methods

### Participants

Participants were recruited from cohorts within the Wisconsin Alzheimer’s Disease Research Center (ADRC) including the Investigating Memory in Preclinical Alzheimer’s Disease–Causes and Treatments (IMPACT) cohort and the Healthy Older Controls cohort. All participants included in this study were considered cognitively unimpaired at the time of enrollment and have been described in detail previously [29,30]. In addition, a portion of these results have been reported in the postmenopausal females who participated in this study [31]. The recruitment period started on April 6^th^, 2018, and ended on August 28^th^, 2019. All study procedures were approved by the University of Wisconsin–Madison Institutional Review Board and performed according to the Declaration of Helsinki by obtaining written informed consent with signed consent documentation from each participant.

Ninety-five cognitively unimpaired adults (32 males, 63 females) between 55–69 years of age participated in this study. Females were postmenopausal for greater than 1 year and were not currently taking oral menopausal hormone therapy. Exclusion criteria consisted of a confirmed diagnosis of MCI or dementia of any kind, body mass index greater than 34.9 kg/m^2^, uncontrolled hypertension, significant surgical history, history of clinically significant stroke, cerebrovascular disease, or other major neurological disorders. Participants with controlled hypertension, defined as taking prescribed blood pressure (BP) medication to manage their BP were included in the study. If a participant reported to have hypertension and was not taking any anti-hypertensive medication, they were excluded. Initial study inclusion and exclusion was determined by a phone screening.

### Experimental procedures

Participants visited the Bruno Balke Biodynamics Laboratory at the University of Wisconsin-Madison on two separate occasions for a screen day and experimental study day. On a separate day, participants visited the Wisconsin Institutes for Medical Research in Madison, WI for an MRI scan. On the experimental study day, participants arrived at the laboratory after a 4-hour fast and refrained from performing strenuous exercise during the previous 24 hours. Participants were instructed to avoid consumption of caffeine or chocolate during the previous 24 hours, alcohol during the previous 24 hours, and aspirin or non-steroidal anti-inflammatory drugs during the previous 48 hours prior to the experimental study day visit. Participants were asked to withhold over-the-counter medications on the experimental study day. This purpose of withholding over-the-counter medications and refraining from exercise, caffeine or chocolate was to minimize the acute impact of these substances on cerebral blood flow regulation. All experimental study day procedures were performed while participants laid supine in a dimly lit, temperature-controlled room kept between 22–24°C and all study personnel were blinded to *APOE*ε4 status. All experimental study day visits and MRI scans took place prior to the COVID-19 pandemic.

### Screen day measurements

The screen day visit took approximately 1 hour. Upon arrival at the laboratory, height and weight were obtained using a standard scale and stadiometer. The screen day visit consisted of a brief familiarization with study procedures, a supine arterial BP measurement in triplicate using a brachial cuff (Datex Ohmeda, GE Healthcare, Fairfield, CT, United States) after resting quietly in a dimly lit room for 10 min, and a middle cerebral artery (MCA) screening using transcranial Doppler (TCD) ultrasound. The angle of the TCD ultrasound probe, anatomical location, depth of the signal, and mean velocity were recorded to be used as a reference on the experimental study day to ensure repeatability of measurements. Participants were familiarized with the CVR protocol and the *n*-back working memory test (*n*-back).

### Experimental study day measurements

The experimental study day measurements took approximately 2 hours. Participants remained supine during instrumentation and throughout the entire protocol. An overview of the experimental study day protocol is as follows: instrumentation, baseline hemodynamic measurements, and middle cerebral artery velocity (MCAv) signal acquisition. This was followed by neurovascular coupling assessments, a 10 min washout period, and stepped hypercapnia assessments. Following 10 min of supine rest, arterial BP was measured in triplicate using a brachial BP cuff and the average value was recorded. Heart rate (HR) was measured using a 3-lead electrocardiogram (CardioCap, Datex Ohmeda, GE Healthcare, Fairfield, CT, United States). Mean arterial blood pressure (MAP) was continuously measured using a non-invasive finger cuff (NOVA, Finapres Medical Systems, Amsterdam, The Netherlands). End-tidal CO_2_ (ETCO_2_) was measured using a nasal cannula. MCAv of the left MCA was measured using a 2-MHz TCD ultrasound probe (Spencer Technologies, Seattle, WA, United States). Quality MCAv signals were obtained using established guidelines [32] and signal quality was confirmed prior to and throughout hypercapnia and the *n*-back test.

### Experimental study day protocol

NVC was assessed by measuring the percent change in MCAv in response to the *n*-back test. Participants completed a 3 min *n*-back test, a test of working memory [33], as previously described [34]. Briefly, participants were asked to identify if the object on the screen was the same object as two slides ago. Participants were instructed to perform the *n*-back test to the best of their abilities.

A 10 min washout period was observed between the CVR protocol and NVC protocol. Participants were fitted with a mask covering their nose and mouth with a one-way valve to prevent re-breathing (7450-V2, Hans Rudolph, Inc. Shawnee, KS, United States). Participants completed a stepped hypercapnia protocol as previously described [19,31]. Briefly, participants breathed normocapnic room air for 5 min followed by stepwise elevations of hypercapnic air at 2%, 4%, and 6% inspired CO_2_ for 5 min each.

### Apolipoprotein E genotyping

Apolipoprotein status was determined using competitive allele-specific polymerase chain reaction-based genotyping assays (LGC Genomics, Beverly, MA) [29]. Participants were considered *APOE*ε4+ if they had one or more copies of the ε4 allele (i.e., *APOE* ε2/ε4, ε3/ε4, or, ε4/ε4). Participants were considered *APOE*ε4- if they did not have one or more copies of the ε4 allele (i.e., *APOE* ε2/ε2, ε2/ε3, or ε3/ε3).

### MRI measurements

On a separate visit from the cerebrovascular function testing, MRI brain scans were completed on a 3T clinical MRI scanner (GE Healthcare, Waukesha, WI, United States) at the Wisconsin Institutes for Medical Research. The MRI scan took approximately 1 hour. Participants remained supine in the MRI scanner throughout the entire protocol. A brief overview of the MRI measurement protocol is as follows: instrumentation, brain volume scan, microvascular cerebral perfusion scan, and macrovascular blood flow scan. Brain volumes were measured using a T1-weighted structural brain volume (BRAVO) scan with the following scan parameters: fast spoiled gradient echo sequence, inversion time = 450 ms, repetition time = 8.1 ms, echo time = 3.2 ms, flip angle = 12°, acquisition matrix = 256 × 256, field of view (FOV) = 256 mm, slice thickness = 1.0 mm, and scan time ∼8 min.

Microvascular cerebral prefusion was measured using background-suppressed pseudocontinuous arterial spin labeling (ASL) MRI utilizing a 3D fast spin-echo stack of spiral sequence described previously [12,35] with the following scan parameters: echo spacing  = 4.9 ms, TE  =  10.5 ms with centric phase encoding, spiral arms  =  8, spiral readout duration  = 4 ms, FOV  = 240 × 240 × 176 mm, 4 mm isotropic spatial resolution, reconstructed matrix size  =  128 × 128 × 44, number of averages (NEX)  =  3, labeling RF amplitude  = 0.24 mG, and scan time  ~4.5 min. Post-labeling delay was 2025 ms for all scans. Immediately after each ASL scan, a proton density (PD) reference scan was performed with identical imaging acquisition parameters without ASL labeling but with a saturation pulse applied 2.0 seconds prior to imaging. This PD image was used for ASL flow quantification as well as for image registration.

Macrovascular blood flow of the large intracranial vessels was measured using 4D flow MRI using a 3D radially undersampled sequence (PC-VIPR) as previously described [36,37] with the following scan parameters: velocity encoding (Venc) = 80cm/s, imaging volume = 220 mm x 220 mm x 160 mm, acquired isotropic spatial resolution = 0.7mm×0.7mm×0.7mm, TR = 7.8ms, TE = 2.7ms, flip angle = 8°, bandwidth = 83.3kHz, 14,000 projection angles, and scan time ∼7min.

### CVR and NVC data analysis

Cardiovascular and cerebrovascular variables were recorded using LabChart 8 at 250 Hz (AD Instruments, Dunedin, New Zealand) and stored offline for analysis. CVR was calculated as the linear relationship between the percent change in ETCO_2_ and the percent change in MCAv in response to hypercapnia [31]. NVC was calculated as the percent change in MCAv in response to the *n*-back test as previously described [34].

### MRI data analysis

Individual structural MRI scans were segmented in Statistical Parametric Mapping version 12 (SPM12) into gray matter, white matter, and cerebrospinal fluid [38]. Total brain volume was calculated as the sum of gray and white matter volumes. Intracranial volume was calculated as the sum of gray matter, white matter, and cerebrospinal fluid volumes.

4D flow MRI scans were analyzed as previously described [21]. Time-resolved velocity and magnitude data were reconstructed offline by retrospectively gating into 20 cardiac phases using temporal interpolation [39]. Flow was calculated from the velocity and diameter measurements for each of the 20 cardiac phases. All scans underwent background phase offset correction, eddy current correction, and automatic phase unwrapping to minimize potential for velocity aliasing [40]. Individual vessel segmentation of left and right internal carotid arteries (ICA), left and right MCAs, and basilar artery were performed in MATLAB using an in-house tool for semi-automated cerebrovascular flow analysis [41]. Blood flow was averaged along the length of each vessel. The ICAs were measured below the carotid siphon along the cervical and petrous portions. The MCAs were measured at the M1 segment. The basilar artery was measured above the bifurcation of the vertebral arteries and below the superior cerebellar artery. Total CBF through the major arteries was calculated as the sum of the left and right ICAs and the basilar artery [42].

In addition to 4D flow CBF data, cerebral perfusion data were included to evaluate microvascular gray matter perfusion. Cerebral perfusion data were extracted from pseudocontinuous ASL cerebral perfusion images using SPM12 as previously described [12]. Each participant’s PD image was first registered to the T1 image and the derived transformation matrix was applied to the average quantitative cerebral perfusion map. With resampling to a 2 x 2 x 2 mm^3^ voxel size, the T1 volume and associated cerebral perfusion image were subsequently spatially normalized to the Montreal Neurological Institute (MNI) template. The normalized cerebral perfusion maps were then smoothed using an 8-mm full-width at half-maximum Gaussian kernel. Total gray matter cerebral perfusion was analyzed and reported.

### Statistical analysis

Normality of all variables was assessed using Shapiro-Wilk tests and visually inspected using histograms and QQ plots. Equal variance of all variables was assessed using Levene’s test. Participant characteristics, cardiovascular variables at rest, brain volumes, blood flow through each artery, and cerebral perfusion were compared between *APOE*ε4+ and *APOE*ε4- adults using independent samples t-tests (two-tailed). Cardiovascular and cerebrovascular variables at baseline, in response to hypercapnia, and in response to the *n*-back test were compared between *APOE*ε4+ and *APOE*ε4- adults using independent samples t-tests (two-tailed). In the event of unequal variance between *APOE*ε4+ and *APOE*ε4- adults, Welch’s t-tests were used. Effect sizes for all comparisons between groups were calculated using Cohen’s *d* interpreted as a small (*d* = 0.2), medium (*d* = 0.5), or large (*d* = 0.8) effect [43]. The relationship between total CBF measured using 4D flow MRI and cerebral perfusion measured using ASL was assessed using a Pearson correlation. All statistical analyses were completed using R software. The mean ± standard deviation for all variables is presented. Statistical significance was set a priori at *P* < 0.05.

## Results

### Participants

Out of the initial 95 participants recruited for this study, eight participants were excluded from the final analysis due to inadequate data quality (MAP or MCAv signal loss; n = 7) or incomplete *APOE* genotype data (n = 1). Of the remaining 87 participants, 37 participants were *APOE*ε4+ and 50 participants were *APOE*ε4-. Participant characteristics and cardiovascular variables at rest are presented in Table 1. There were no differences in participant characteristics and cardiovascular variables at rest between *APOE*ε4+ and *APOE*ε4- adults (Table 1). Within the *APOE*ε4+ group, four participants were ε2/ε4, 28 participants were ε3/ε4, and five participants were ε4/ε4. Within the *APOE*ε4- group, 11 participants were ε2/ε3 and 39 participants were ε3/ε3.

**Table 1:** Participant characteristics and selected cardiovascular variables at rest.

Variable	*APOE*ε4+			*APOE*ε4-			*P*-value (Effect Size)
n		37			50		
Sex (M/F)	13 / 24			14 / 36			
Age (y)	63	±	4	63	±	4	0.848 (0.042)
Ethnicity							
Hispanic		0			1		
Non-Hispanic		37			49		
Race							
African American		1			0		
Asian		0			1		
White		36			49		
Education (y)	17	±	2	17	±	3	0.507 (0.145)
Height (cm)	171	±	9	168	±	8	0.143 (0.321)
Weight (kg)	78	±	15	76	±	16	0.553 (0.129)
Body Mass Index (kg/m^2^)	27	±	4	27	±	4	0.854 (0.040)
HR (bpm)	60	±	9	60	±	8	0.907 (0.025)
Systolic BP (mmHg)	127	±	17	125	±	14	0.599 (0.115)
Diastolic BP (mmHg)	76	±	8	75	±	8	0.385 (0.189)
Mean Arterial BP (mmHg)	93	±	10	92	±	9	0.457 (0.162)
MoCA	28	±	2	28	±	2	0.684 (0.089)
Family History of Dementia (n, %)	26, 70			34, 68			
Controlled Hypertension (n, %)	6, 16			12, 24			

Values expressed as mean ± SD. *APOE*, apolipoprotein; BP, blood pressure; HR, heart rate; MoCA, Montreal Cognitive Assessment. *P*-value indicates result of independent samples t-test comparing *APOE*ε4 positive (*APOE*ε4+) and *APOE*ε4 negative (*APOE*ε4-) adults. Effect size calculated using Cohen’s *d*.

### Brain volumes

There were no differences in gray matter volume, white matter volume, total brain volume, cerebrospinal fluid, or intracranial volume between *APOE*ε4+ and *APOE*ε4- adults (Table 2).

**Table 2:** Brain volumes between *APOE*ε4+ and *APOE*ε4- adults.

Variable	*APOE*ε4+ n = 37			*APOE*ε4- n = 50			*P*-value (Effect Size)
Gray Matter (L)	0.7	±	0.1	0.7	±	0.1	0.203 (0.278)
White Matter (L)	0.4	±	0.1	0.4	±	0.1	0.165 (0.304)
Total Brain Volume (L)	1.1	±	0.1	1.1	±	0.1	0.151 (0.315)
Cerebrospinal Fluid (L)	0.3	±	0.1	0.3	±	0.1	0.868 (0.036)
Intracranial Volume (L)	1.5	±	0.1	1.4	±	0.1	0.262 (0.245)

Values expressed as mean ± SD. APOE, apolipoprotein. Total brain volume was calculated as the sum of gray and white matter volumes. Intracranial volume was calculated as the sum of gray matter, white matter, and cerebrospinal fluid. P-value indicates result of independent samples t-test comparing APOEε4 positive (APOEε4+, n = 37) and APOEε4 negative (APOEε4-, n = 50) adults. Effect size calculated using Cohen’s d.

### Blood flow through the intracranial arteries

**Fig 1:**
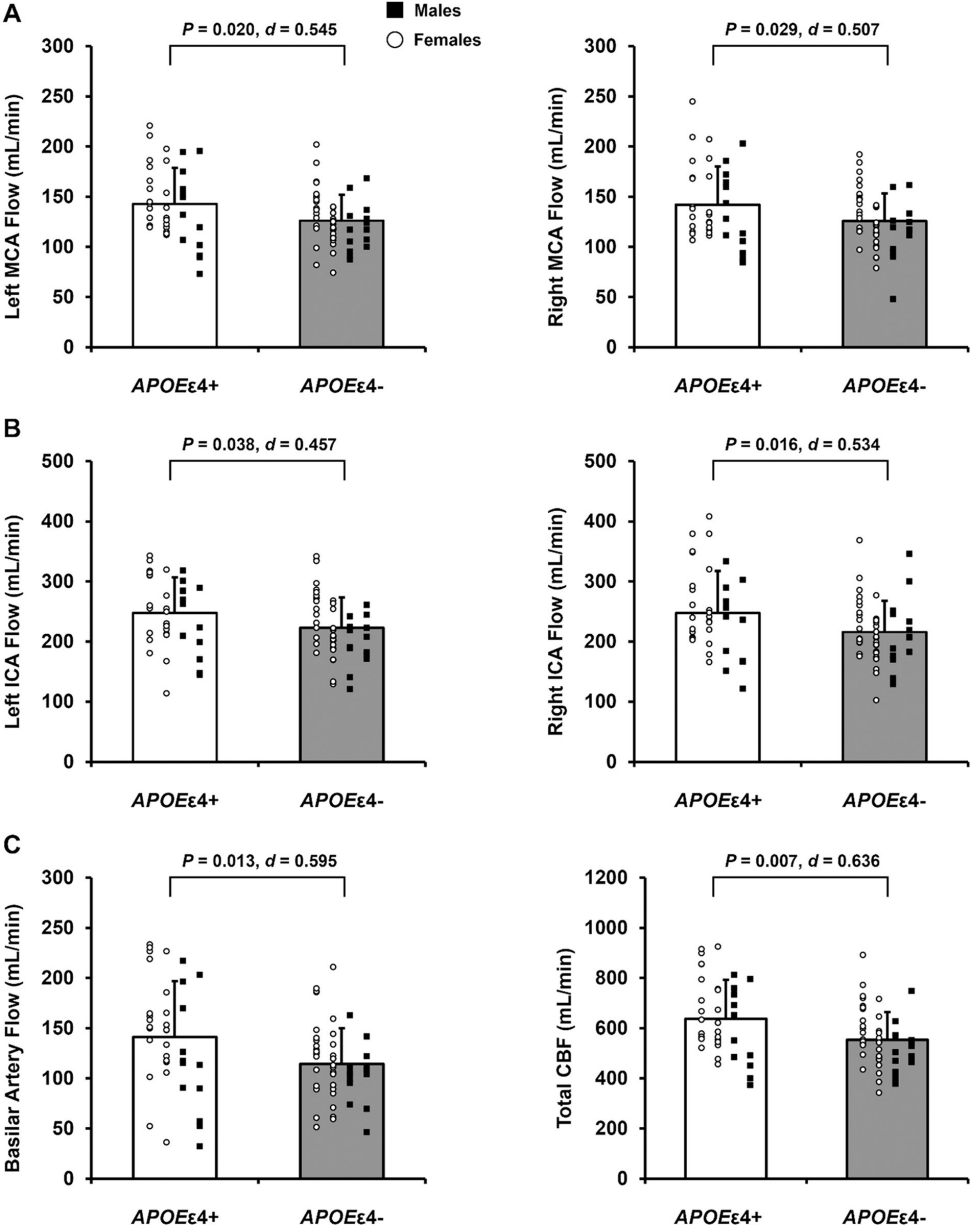
Blood flow through the intracranial arteries in apolipoprotein ε4 positive (*APOE*ε4+) and apolipoprotein ε4 negative (*APOE*ε4-) adults. Data expressed as individual data and means ± standard deviation. *APOE*ε4+ means represented by white bars and *APOE*ε4- means represented by gray bars. Within each group, females are represented as white circles and males are represented as black squares. A: Middle cerebral artery (MCA) blood flow. B: Internal carotid artery (ICA) blood flow. C: Basilar artery blood flow and total cerebral blood flow (CBF). *P*-value indicates result of independent samples t-test comparing blood flow in each artery between *APOE*ε4+ (n = 37) and *APOE*ε4- (n = 50) adults*. Effect size calculated as Cohen’s *d*. *Note: For right MCA flow, n = 37 for *APOE*ε4+ and n = 49 for *APOE*ε4-.

Blood flow through the left and right MCA (Fig 1A), left and right ICA (Fig 1B), basilar artery (Fig 1C, left panel), and total CBF (Fig 1C, right panel) was greater in *APOE*ε4+ compared with *APOE*ε4- adults.

### Cerebral perfusion

**Fig 2:**
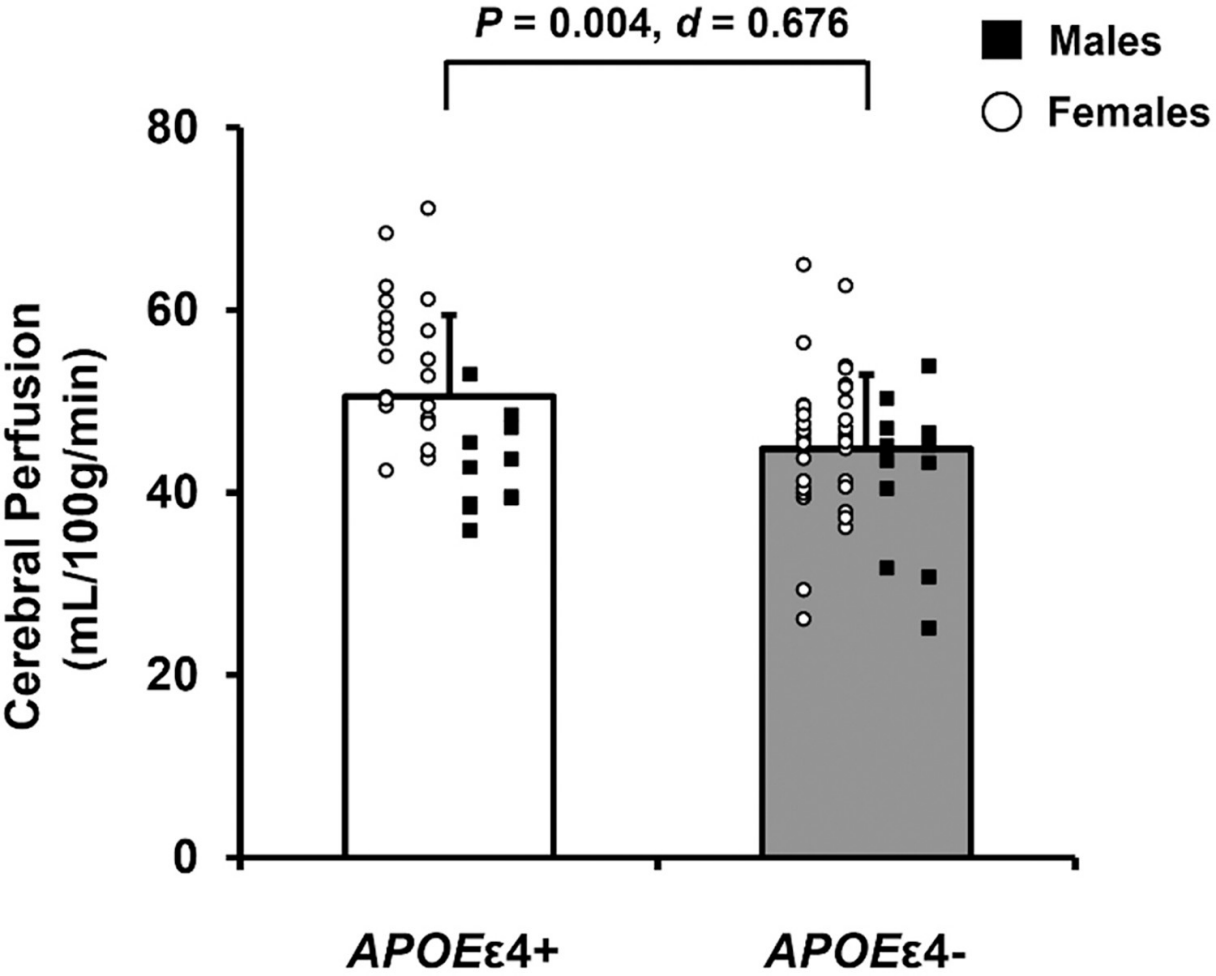
Microvascular cerebral perfusion in apolipoprotein ε4 positive (*APOE*ε4+) and apolipoprotein ε4 negative (*APOE*ε4-) adults. Data expressed as individual data and means ± standard deviation. *APOE*ε4+ means represented by white bars and *APOE*ε4- means represented by gray bars. Within each group, females are represented as white circles and males are represented as black squares. *P*-value indicates result of independent samples t-test comparing cerebral perfusion between *APOE*ε4+ (n = 32) and *APOE*ε4- (n = 47) adults. Effect size calculated as Cohen’s *d*.

Five *APOE*ε4+ and three *APOE*ε4- adults were excluded from the cerebral perfusion analysis due to incomplete data (n = 6) or incompatible inversion times for participant specific flow (n = 2). In the remaining 79 participants, cerebral perfusion was greater in *APOE*ε4+ compared with *APOE*ε4- adults (Fig 2).

### CVR

**Fig 3:**
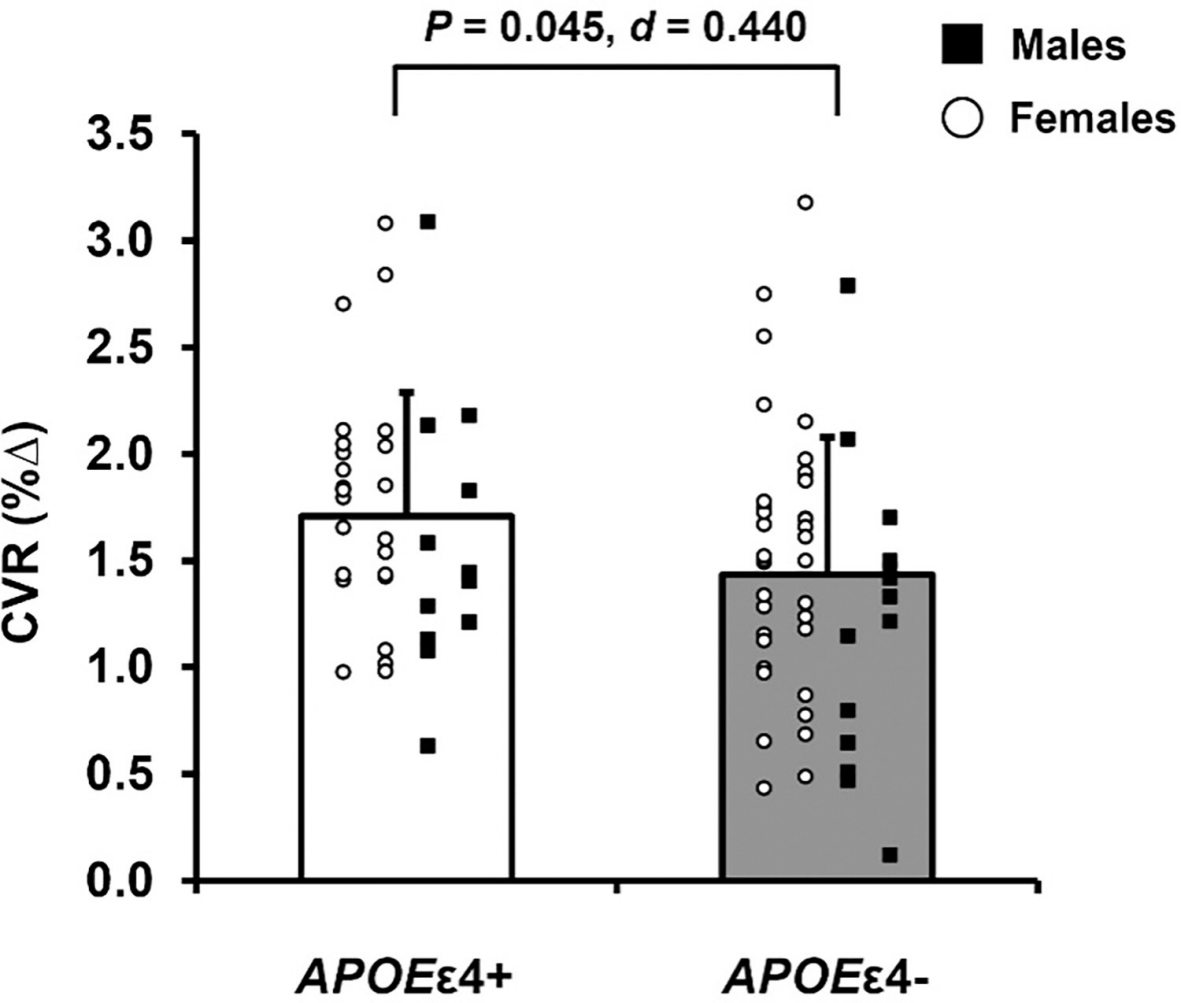
Cerebrovascular reactivity (CVR) to hypercapnia in apolipoprotein ε4 positive (*APOE*ε4+) and apolipoprotein ε4 negative (*APOE*ε4-) adults. Data expressed as individual data and means ± standard deviation. *APOE*ε4+ means represented by white bars and *APOE*ε4- means represented by gray bars. Within each group, females are represented as white circles and males are represented as black squares. CVR expressed as the linear relationship between the percent change from baseline in middle cerebral artery blood velocity and the percent change in end-tidal CO_2_. *P*-value indicates result of independent samples t-test comparing CVR to hypercapnia between *APOE*ε4+ (n = 37) and *APOE*ε4- (n = 50) adults. Effect size calculated as Cohen’s *d*.

*APOE*ε4+ adults had greater CVR compared with *APOE*ε4- adults (Fig 3). Importantly, there were no differences in HR, MAP, or MCAv at baseline during room air or in response to hypercapnia between *APOE*ε4+ and *APOE*ε4- adults (S1 Table).

### NVC

**Fig 4:**
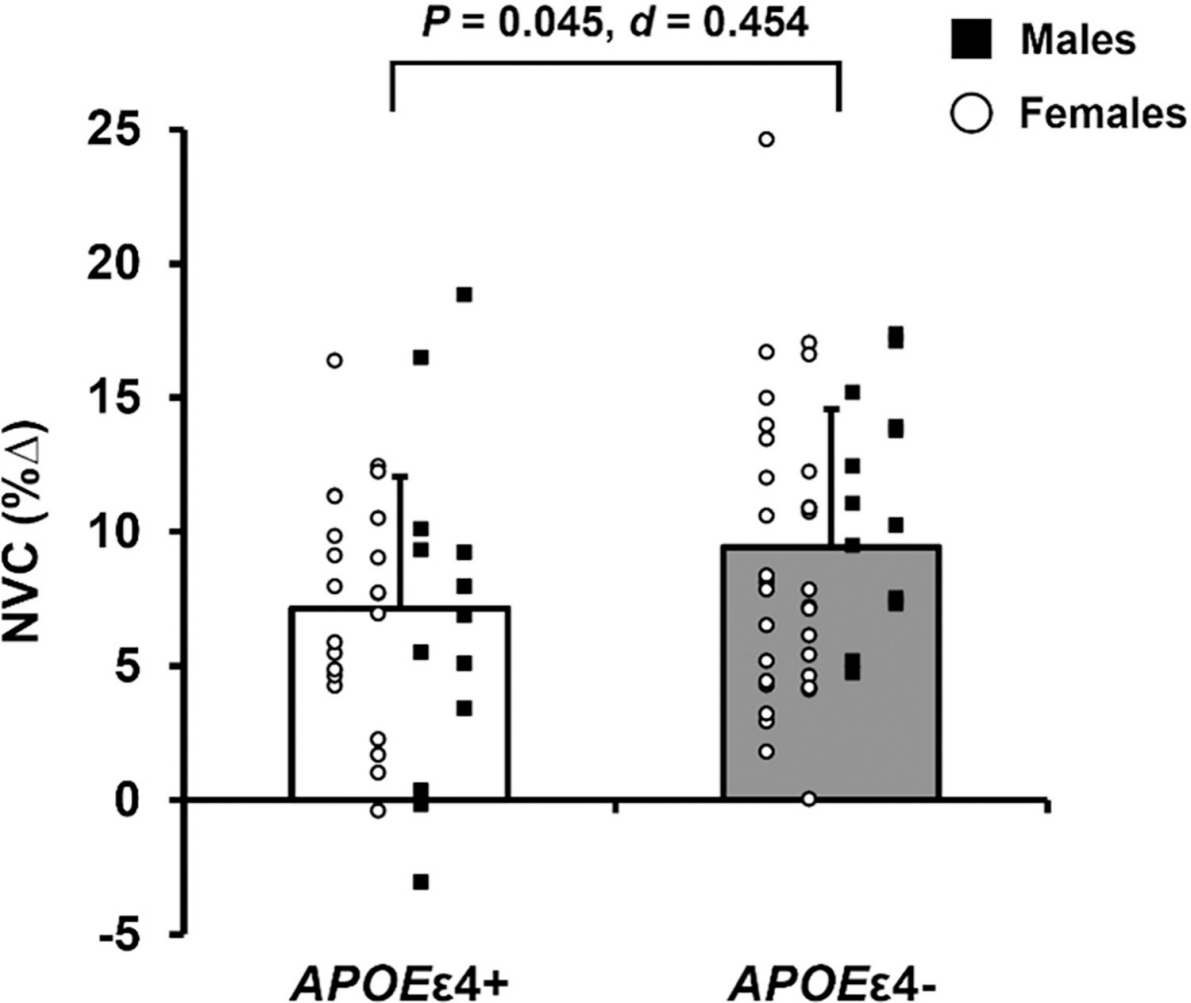
Neurovascular coupling (NVC) in response to the *n*-back working memory test in apolipoprotein ε4 positive (*APOE*ε4+) and apolipoprotein ε4 negative (*APOE*ε4-) adults. Data expressed as individual data and means ± standard deviation. *APOE*ε4+ means represented by white bars and *APOE*ε4- means represented by gray bars. Within each group, females are represented as white circles and males are represented as black squares. NVC is expressed as the percent change from baseline in middle cerebral artery blood velocity. *P*-value indicates result of independent samples t-test comparing NVC in response to the *n*-back test between *APOE*ε4+ (n = 36) and *APOE*ε4- (n = 46) adults. Effect size calculated as Cohen’s *d*.

One *APOE*ε4+ and four *APOE*ε4- adults were excluded from the *n*-back analysis due to inadequate data quality (MAP or MCAv signal loss). In the remaining 82 participants NVC, expressed as the percent change in MCAv in response to the *n*-back test, was lower in *APOE*ε4+ compared with *APOE*ε4- adults (Fig 4). There were no differences in HR, MAP, or MCAv at baseline or in response to the *n*-back test between *APOE*ε4+ and *APOE*ε4- adults (S2 Table).

## Discussion

The primary findings of this study were that *APOE*ε4+ adults had greater CBF at rest and CVR, yet demonstrated lower NVC responses, compared with *APOE*ε4- adults. Collectively, the novel findings of this study suggest that despite greater CBF at rest, impaired NVC, indicated by a blunted MCAv response to the *n*-back working memory test, may occur early in the time course of declines in cognitive function in cognitively unimpaired adult *APOE*ε4 carriers.

Consistent with previous research, we report greater MCA blood flow at rest in *APOE*ε4+ compared with *APOE*ε4- adults [11]. Unique to the present study is the inclusion of blood flow through the ICAs, basilar artery, and total CBF using 4D flow MRI. These data provide insight into intracranial CBF supply and, with the addition of ASL data, microvascular cerebral perfusion. In support of previous research [11,14–16], we report greater total CBF and cerebral perfusion at rest in *APOE*ε4+ compared with *APOE*ε4- adults, despite no differences in brain volumes. These findings add to existing evidence supporting the cerebrovascular compensation hypothesis suggesting that *APOE*ε4+ adults have greater CBF and gray matter perfusion to compensate for the detrimental effects of the *APOE*ε4 allele across the lifespan until a hemodynamic breaking point at which rapid declines in CBF and cerebral perfusion occur. Indeed, cerebral perfusion is lower in older *APOE*ε4+ adults (74 ± 7 years) compared with *APOE*ε4- older adults [13] suggesting that a rapid decline in cerebral perfusion occurs in older age. For example, an ~8-year longitudinal study in cognitively unimpaired older adults (69 ± 7 years) found that declines in cerebral perfusion were greater in *APOE*ε4+ compared with *APOE*ε4- older adults [16]. As participants recruited for the present study were younger (62 ± 4 years), generally healthy, and cognitively unimpaired, the findings of this study provide insight into a critical period where clinically relevant declines in total CBF and microvascular cerebral perfusion likely begin or are occurring.

A novel aspect of the present study is the inclusion of cerebrovascular responses to both chemical and cognitive stimuli. Importantly, these tests of cerebrovascular function may provide insight into cerebrovascular health, especially in aging adults who do not demonstrate evidence of cognitive dysfunction. Declines in CVR to chemical stimuli such as hypercapnia occur across the lifespan [8,18]. Longitudinal data suggest that attenuated CVR is associated with greater declines in cognitive performance and increased risk of AD, with greater rates of decline in *APOE*ε4+ adults [44]. To our knowledge, the present study is the first to evaluate the effect of the *APOE*ε4 allele on CVR to hypercapnia in cognitively unimpaired, healthy adults 55–69 years of age. Our finding that *APOE*ε4+ adults have greater CVR compared with *APOE*ε4- adults is opposite of our original hypothesis and in conflict with previous work on *APOE*ε4 and CVR [23,24]. However, the previous studies evaluated only young (24 ± 5 years) [23] or older adults (78 ± 5 years) [24]. Although CVR was lower in *APOE*ε4+ young adults, CVR of the hippocampi was evaluated using BOLD MRI which reflects changes in deoxyhemoglobin of the left and right hippocampus. While Hajjar et al. 2015 utilized techniques similar to the present study, participants were older, had a history of stroke and cardiovascular disease, and the majority (74%) were hypertensive [24], which limits the applicability of these findings in the context of healthy aging. Therefore, it is possible that methodological and age group differences could explain the conflicting findings. Moreover, augmented CVR in *APOE*ε4+ adults observed in the present study (in addition to greater total CBF) supports the proposed cerebrovascular compensation hypothesis such that responses to chemical stimuli may be amplified before declines start to occur.

CBF responses to acute cognitive activity are indicative of day-to-day tasks which elicit an increase in neuronal activity and metabolic demand of the brain. Impairments in NVC in the form of attenuated BOLD MRI and cerebral artery blood velocity responses to increased metabolic demand of the brain occur with advancing age [20,45] and impaired NVC may be an early indication of vascular dysfunction [26]. *APOE*ε4 modulates cerebrovascular function directly at the neurovascular unit and indirectly through its effect on the peripheral and cerebral vasculature, ultimately contributing to cognitive decline [17]. In the present study, *APOE*ε4+ adults demonstrated blunted MCAv responses to an increase in metabolic demand of the brain during the *n*-back test. These findings suggest that *APOE*ε4+ adults have impaired NVC in response to a test of working memory. Previous work reported that the BOLD signal change in response to a memory task is greater in *APOE*ε4+ relative to *APOE*ε4- young adults [27]. In a follow-up study that included aging adults (64 ± 7 years), presence of the *APOE*ε4 allele had the opposite effect such that the BOLD response to a memory task was lower in *APOE*ε4+ relative to *APOE*ε4- adults [14]. Collectively, the Filippini et al. 2011 and 2009 studies suggest a biphasic effect of *APOE*ε4 on NVC responses to a memory task, similar to the biphasic patterns reported with CBF and cerebral perfusion. However, the current literature on the effect of *APOE*ε4 on BOLD responses to acute cognitive activity in aging adults remains unclear [28]. In agreement with the findings of the present study, Fleisher et al. 2009 report attenuated BOLD responses to cognitive activity [46], while other studies suggest greater BOLD signal responses [47,48] or no difference between *APOE*ε4 carriers and non-carriers [49]. Conflicts in the literature are likely due to the broad age range of participants, differences in the overall health and cognitive status of participants, as well as methodological differences between studies. In addition, the mechanisms by which cerebral blood velocity responses to chemical stimuli are augmented but responses to cognitive stimuli are impaired in cognitively unimpaired *APOE*ε4 carriers are unknown.

Alternatively, it is possible that lower NVC and a blunted MCAv response to acute cognitive activity could be interpreted as a lesser need for blood flow to match an increase in metabolic demand of the brain. This is a plausible explanation given the observed greater total CBF and cerebral perfusion in *APOE*ε4+ compared with *APOE*ε4- adults in the present study; however, there were no differences in MCAv at rest between groups. Measurement of MCAv via TCD ultrasound is considered an estimate of flow because it does not include vessel diameter. Therefore, we cannot completely rule out that possibility that changes in MCA vessel diameter occurred in response to hypercapnia and acute cognitive activity. However, previous studies investigating MCA diameter changes in response to hypercapnia suggest that changes in the diameter of the MCA is minimal in older adults [21,50]. Therefore, this likely does not influence the results of the present study.

A better understanding of the underlying mechanisms that contribute to vascular dysfunction in AD and identification of novel, noninvasive biomarkers of vascular dysfunction in the context of cognitive and neurological impairments have been identified as two critical areas to move the field forward [51]. In support of the latter, impaired NVC may serve as a noninvasive, early indication of vascular dysfunction in individuals at genetic predisposition to develop AD [52]. In a healthy brain, the neurovascular unit works to direct blood flow to regions of the brain with higher metabolic activity (i.e. neurovascular coupling). However, if blood flow does not match neuronal activation and metabolic need, there could be cognitive consequences. Indeed, neurovascular coupling is disrupted in multiple neurological conditions including Alzheimer’s disease [4,5]. *APOE* genotype differentially modulates the cerebral and peripheral vasculature, and as a result, may affect cerebrovascular function [17]. For example, compared with cognitively unimpaired *APOE*ε4- middle-aged adults, *APOE*ε4+ adults have greater transcranial pulse wave velocity despite no difference in CBF which is indicative of vascular stiffening within the cerebrovasculature [53]. In mouse models, *APOE*ε4+ mice have lower CBF, impaired NVC, disrupted white matter integrity, and exhibit cognitive dysfunction compared with *APOE*ε4- mice [54]. Further, *APOE*ε4 expression may impede amyloid beta clearance leading to amyloid beta deposition [55]. In humans, *APOE*ε4 exacerbates the detrimental effects of cardiovascular disease risk factors such as hypertension and hypercholesterolemia [56] and disrupts the blood brain barrier [57]. Taken together, *APOE*ε4 affects the cerebral and peripheral vasculature, potentially beginning at an early age and exerting its effect throughout the lifespan, resulting in compensatory mechanisms, such as augmented CBF and CVR to offset the detrimental effects of *APOE*ε4.

There are several limitations of the present study. This study includes participants that are relatively healthy in attempt to isolate the effect of APOE genotype. The participants were also primarily white and well-educated; thus, this sample may not be representative of the general population. Some participants reported they were prescribed common medications such as blood pressure medication, statins, metformin, thyroid medication, or medication for depression or anxiety. The impact of chronic medication use on cerebral blood flow warrants further investigation. As mentioned above, measurement of MCAv via TCD ultrasound is considered an estimate of flow; however, use of TCD ultrasound enhances temporal resolution by evaluating beat-to-beat changes in MCAv which provides insight into rapid changes in CBF. Future studies may assess the influence of *APOE*ε4+ on cerebrovascular function in other cerebral arteries besides the MCA. To quantify NVC, we used the *n*-back test, a cognitive test of working memory, but the test was not scored due to the experimental setup and instrumentation. Participants were instructed to complete the test to the best of their ability and were not informed about the purpose of completing the test until the conclusion of the experimental study visit. We cannot assess the “dose” of the NVC task based on perceived difficulty of the task. However, this is why NVC was calculated as a percent change in blood velocity from baseline. Future studies may also assess the interaction of sex, *APOE* genotype (ε2/ε4, ε3/ε3, etc.), and/or other genetic or environmental factors on cerebral blood flow regulation. We grouped all the *APOE*ε4+ carriers together. However, within *APOE*ε4+ carriers, different genotypes (for example ε2/ε4 vs. ε3/ε4) may have differential impacts cerebrovascular regulation and subsequent AD risk. Lastly, MRI data used for this study were collected as part of the ongoing longitudinal Wisconsin ADRC research study on early detection of AD [29]. The average time between MRI scan and the experimental study day visit was 1.8 ± 1.4 years, with MRI scans occurring either before or after the experimental study day visit depending on the participant.

In summary, despite no differences in brain volumes, cognitively unimpaired *APOE*ε4+ adults had greater CBF through the large intracranial arteries, microvascular cerebral perfusion, and CVR to hypercapnia compared with *APOE*ε4- adults 55–69 years of age. Importantly, *APOE*ε4+ adults demonstrated lower cerebral blood velocity responses to a cognitive test suggesting impaired NVC. These findings suggest that impairments in NVC may occur early in the time course of declines in cognitive function in adult *APOE*ε4+ carriers. Functional measures of cerebrovascular health may be a useful diagnostic tool in determining future risk of AD in individuals at genetic predisposition to develop AD.

## Supporting information

S1 Table Cardiovascular and cerebrovascular variables at baseline and in response to hypercapnia between *APOE*ε4+ and *APOE*ε4- adults. Values expressed as mean ± SD. *APOE*, apolipoprotein; MAP, mean arterial blood pressure; MCAv, middle cerebral artery blood velocity. *P*-value indicates result of independent samples t-test comparing *APOE*ε4 positive (*APOE*ε4+, n = 37) and *APOE*ε4 negative (*APOE*ε4-, n = 50) adults during room air and in response to 6% CO_2_. Effect size calculated using Cohen’s *d*.
(DOCX)

S2 Table Cardiovascular and cerebrovascular variables at baseline and in response to the *n*-back working memory test between *APOE*ε4+ and *APOE*ε4- adults. Values expressed as mean ± SD. *APOE*, apolipoprotein; MAP, mean arterial blood pressure; MCAv, middle cerebral artery blood velocity. *P*-value and effect size in the column indicates result of independent samples t-test comparing *APOE*ε4 positive (*APOE*ε4+, n = 36) and *APOE*ε4 negative (*APOE*ε4-, n = 46) adults at baseline and in response to the *n*-back test. Effect size calculated using Cohen’s *d*.
(DOCX)
