# Supplementary material for: Lower neurovascular coupling response despite higher cerebral blood flow at rest in apolipoprotein ɛ4 positive adults
Source: PLoS One. 2024 Dec 3;19(12):e0314744. doi: 10.1371/journal.pone.0314744 (PMC11614282; doi:10.1371/journal.pone.0314744)
Supplement: S1 Table — Values expressed as mean ± SD. APOE, apolipoprotein; MAP, mean arterial blood pressure; MCAv, middle cerebral artery blood velocity. P-value indicates result of independent samples t-test comparing APOEε4 positive (APOEε4+, n = 37) and APOEε4 negative (APOEε4-, n = 50) adults during room air and in response to 6% CO2. Effect size calculated using Cohen’s d. (DOCX) [file pone.0314744.s001.docx]

| **Variable** | ***APOE*ε4+** | | | ***APOE*ε4-** | | | ***P*-value (Effect Size)** |
| --- | --- | --- | --- | --- | --- | --- | --- |
| Heart Rate (bpm)  Room air  6% CO_2_ | 60  64 | ±  ± | 8  8 | 59  63 | ±  ± | 9  10 | 0.653 (0.098)  0.695 (0.085) |
| MAP (mmHg)  Room air  6% CO_2_ | 102  110 | ±  ± | 12  11 | 101  110 | ±  ± | 13  13 | 0.744 (0.071)  0.752 (0.069) |
| MCAv (cm/s)  Room air  6% CO_2_ | 59  78 | ±  ± | 11  13 | 57  73 | ±  ± | 13  16 | 0.360 (0.200)  0.111 (0.349) |

**S1 Table.** **Cardiovascular and cerebrovascular variables at baseline and in response to hypercapnia between *APOE*ε4+ and *APOE*ε4- adults.**

Values expressed as mean ± SD. *APOE*, apolipoprotein; MAP, mean arterial blood pressure; MCAv, middle cerebral artery blood velocity. *P*-value indicates result of independent samples t-test comparing *APOE*ε4 positive (*APOE*ε4+, n = 37) and *A POE*ε4 negative (*APOE*ε4-, n = 50) adults during room air and in response to 6% CO_2_. Effect size calculated using Cohen’s *d*.
