# Supplementary material for: Lower neurovascular coupling response despite higher cerebral blood flow at rest in apolipoprotein ɛ4 positive adults
Source: PLoS One. 2024 Dec 3;19(12):e0314744. doi: 10.1371/journal.pone.0314744 (PMC11614282; doi:10.1371/journal.pone.0314744)
Supplement: S2 Table — Values expressed as mean ± SD. APOE, apolipoprotein; MAP, mean arterial blood pressure; MCAv, middle cerebral artery blood velocity. P-value and effect size in the column indicates result of independent samples t-test comparing APOEε4 positive (APOEε4+, n = 36) and APOEε4 negative (APOEε4-, n = 46) adults at baseline and in response to the n-back test. Effect size calculated using Cohen’s d. (DOCX) [file pone.0314744.s002.docx]

| **Variable** | ***APOE*ε4+** | | | ***APOE*ε4-** | | | ***P*-value (Effect Size)** |
| --- | --- | --- | --- | --- | --- | --- | --- |
| Heart Rate (bpm)  Baseline  *n*-back | 64  69 | ±  ± | 9  10 | 63  68 | ±  ± | 9  10 | 0.570 (0.127)  0.892 (0.064) |
| MAP (mmHg)  Baseline  *n*-back | 103  107 | ±  ± | 13  13 | 103  106 | ±  ± | 14  14 | 0.945 (0.015)  0.889 (0.042) |
| MCAv (cm/s)  Baseline  *n*-back | 57  61 | ±  ± | 10  11 | 56  61 | ±  ± | 12  13 | 0.700 (0.086)  0.951 (0.026) |

**S2 Table.**  **Cardiovascular and cerebrovascular variables at baseline and in response to the *n*-back working memory test between *APOE*ε4+ and *APOE*ε4- adults.**

Values expressed as mean ± SD. *APOE*, apolipoprotein; MAP, mean arterial blood pressure; MCAv, middle cerebral artery blood velocity. *P*-value and effect size in the column indicates result of independent samples t-test comparing *APOE*ε4 positive (*APOE*ε4+, n = 36) and *APOE*ε4 negative (*APOE*ε4-, n = 46) adults at baseline and in response to the *n*-back test. Effect size calculated using Cohen’s *d*.
